# “The burden is upon your shoulders to feed and take care of your children, not religion or culture”: qualitative evaluation of participatory community dialogues to promote family planning’s holistic benefits and reshape community norms on family success in rural Uganda

**DOI:** 10.1186/s40834-024-00290-y

**Published:** 2024-06-04

**Authors:** Katelyn M. Sileo, Christine Muhumuza, Doreen Tuhebwe, Suyapa Muñoz, Rhoda K. Wanyenze, Trace S. Kershaw, Samuel Sekamatte, Haruna Lule, Susan M. Kiene

**Affiliations:** 1 Department of Public Health, The University of Texas at San Antonio, One UTSA Circle, San Antonio, TX 78249 USA; 2 Department of Epidemiology and Biostatistics, Makerere University School of Public Health, 30A Plot, 30A York Terrace, Kampala, Uganda; 3 Division of Epidemiology and Biostatistics, San Diego State University School of Public Health, 5500 Campanile Dr, San Diego, CA 92182 USA; 4 Department of Health Policy Planning and Management, Makerere University School of Public Health, New Mulago Hill Road, Mulago Kampala, Uganda; 5 Department of Disease Control and Environmental Health, Makerere University School of Public Health, New Mulago Hill Road, Mulago Kampala, Uganda; 6 Department of Social and Behavioral Science, Yale School of Public Health, 60 College Street, New Haven, CT 06510 USA; 7 Butambala District Health Department, Gombe Hospital, Gombe, Uganda; 8 Global Centre of Excellence in Health (GLoCEH), Kampala, Uganda

**Keywords:** Family planning, Contraception, Gender equity, Social norms, Intervention, Uganda

## Abstract

**Background:**

Family planning has significant health and social benefits, but in settings like Uganda, is underutilized due to prevalent community and religious norms promoting large family size and gender inequity. Family Health = Family Wealth (FH = FW) is a multi-level, community-based intervention that used community dialogues grounded in Campbell and Cornish’s social psychological theory of transformative communication to reshape individual endorsement of community norms that negatively affect gender equitable reproductive decision-making among couples in rural Uganda.

**Methods:**

This study aimed to qualitatively evaluate the effect of FH = FW’s community dialogue approach on participants’ personal endorsement of community norms counter to family planning acceptance and gender equity. A pilot quasi-experimental controlled trial was implemented in 2021. This paper uses qualitative, post-intervention data collected from intervention arm participants (*N* = 70) at two time points: 3 weeks post-intervention (in-depth interviews, *n* = 64) and after 10-months follow-up (focus group discussions [*n* = 39] or semi-structured interviews [*n* = 27]). Data were analyzed through thematic analysis.

**Results:**

The community dialogue approach helped couples to reassess community beliefs that reinforce gender inequity and disapproval of family planning. FH = FW’s inclusion of economic and relationship content served as key entry points for couples to discuss family planning. Results are presented in five central themes: (1) Community family size expectations were reconsidered through discussions on economic factors; (2) Showcasing how relationship health and gender equity are central to economic health influenced men’s acceptance of gender equity; (3) Linking relationship health and family planning helped increase positive attitudes towards family planning and the perceived importance of shared household decision-making to family wellness; (4) Program elements to strengthen relationship skills helped to translate gender equitable attitudes into changes in relationship dynamics and to facilitate equitable family planning communication; (5) FH = FW participation increased couples’ collective family planning (and overall health) decision-making and uptake of contraceptive methods.

**Conclusion:**

Community dialogues may be an effective intervention approach to change individual endorsement of widespread community norms that reduce family planning acceptance. Future work should continue to explore innovative ways to use this approach to increase gender equitable reproductive decision-making among couples in settings where gender, religious, and community norms limit reproductive autonomy. Future evaluations of this work should aim to examine change in norms at the community-level.

**Trial registration:**

Clinicaltrials.gov (NCT04262882).

## Introduction

Access to family planning services and contraceptive methods for planning the spacing and timing of pregnancy is a human right and an essential health service with broad health benefits for women and children. By reducing unintended pregnancies, family planning reduces maternal and infant mortality, deaths due to unsafe abortions, and the rate of mother-to-child HIV transmission [1]. It also has positive effects on child nutrition and development by allowing families to invest more in each child [2]. Beyond health, increasing women’s reproductive autonomy is inextricably linked to women’s empowerment and advancing gender equity globally, in part through its positive effect on adolescent girls’ and women’s opportunities for educational and economic advancement, which translates to family and societal-level economic security [2]. Further, when family planning programs are delivered in ways that engage men and reduce utilization barriers related to gender inequity, they can have positive impacts on relationships (e.g., improved communication, equitable decision-making, reduced intimate partner violence) [3–5].

Despite the direct, tangible benefits that family planning has on people’s lives, family planning practitioners and programs may be missing the opportunity to effectively convey the full benefits of family planning to potential users. In resource limited settings, there are considerable barriers to the delivery of person-centered family planning counseling, which is family planning care guided by patients’ needs and preferences, respect for the patient, informed decision-making, and quality provider-patient communication [6–9]. For communities in sub-Saharan Africa, the region with the greatest gaps in family planning coverage globally [10], social, economic, and relationship considerations are often the main motivators for family planning use [11, 12]. Programs that emphasize the economic benefits of family planning have been shown to be especially motivating for men [4, 13] and engaging men in family planning programs is critical to increasing contraceptive uptake in settings with a high unmet need for family planning [14].

Thus, interventions that promote the holistic benefits of family planning tailored to men and women’s individual interests represent a potentially effective approach to increasing informed demand for family planning. However, the success of family planning interventions is likely to be limited if the broader cultural context is not carefully considered in their design and delivery. In many settings with a high-unmet need for family planning, the use of contraceptive methods to space and limit pregnancies are counter to widely held community norms [15]. Research highlights how religious norms and related gender norms shape expectations about family size, men and women’s roles, and polygamy, as well as the strong influence that religious leaders can have on community approval or disapproval of contraceptive use [15–17]. In high fertility settings, social status is often tied to the number of children men and women have (with stigma associated with having few or no children) [18, 19]. High fertility can be driven by the need to have enough children for labor purposes, to be cared for in older age, and to ensure a male child to carry on the family name and property/assets [20, 21]. Further, norms that women should be submissive to their husband, that men are the final decision-makers, and that women and men should not discuss sexual and reproductive health matters are prevalent barriers to family planning [22, 23]. Research reports that community-level gender inequitable norms are negatively associated with contraceptive use [24], and interventions that address these norms, including “gender transformative” approaches aimed to increase gender equitable norms and attitudes, positively influence sexual and reproductive health outcomes, including those related to family planning [3, 5, 25].

In response to these cultural and contextual considerations, we developed Family Health = Family Wealth (FH = FW), a community-based intervention to promote family planning’s holistic benefits through community dialogues aimed to reshape community and gender norms that contribute to a high unmet need for family planning among couples in rural Uganda. Uganda has the seventh highest fertility rate globally (5.45 children per woman in 2021) [26] and 29.7% of married women have an unmet need for family planning (i.e., they want to delay/prevent pregnancy but are not using a modern contraceptive method) [27]. Informed by our earlier, formative work [11, 28], FH = FW was designed to promote family planning’s benefits across three areas of “family health and wealth” to engage both women and men’s interests – physical health, relationship health, and economic health. This cross-cutting theme was a central focus of group discussions grounded in a “community dialogue” approach informed by Campbell and Cornish’s social psychological theory of transformative communication [29]. Dialogues aimed to reshape definitions of “family success” to be inclusive of gender equity and planning for the future, while guiding participants to think critically about broader norms related to cultural beliefs, religion, and gender. The dialogues were enhanced through the inclusion of other multilevel and multicomponent content to address family planning barriers across the social ecological model [30–32].

As reported elsewhere, FH = FW was shown feasible and acceptable, with strong promise in its ability to reduce the unmet need for family planning and affect intermediate outcomes (e.g., couple communication about family planning, gender equitable norms) through a pilot quasi-experimental controlled trial [33, 34]. The objective of the present paper is to qualitatively evaluate the effect of the intervention’s community dialogue approach. We examined participants’ narratives post-intervention for changes in their personal endorsement of community norms that the program aimed to reshape that are counter to family planning acceptance and gender equity, as well as the development of new social norms on definitions of a successful family centered on the holistic benefits of family planning.

## Methods

This study employed a mixed methods embedded experimental design, following Creswell & Plano-Clark [35]. This design included qualitative work to inform the refinement of the intervention [21], followed by a quasi-experimental controlled pilot trial to explore the intervention’s preliminary effects on quantitative outcomes through 10-months follow-up, and two points of post-intervention qualitative follow-up. In this paper, we analyze the post-intervention qualitative data, which was collected to expand on the quantitative findings and provide insight into participants’ experience with the community dialogues and other intervention elements. Conducted from May 2021 to May 2022, the pilot trial compared two matched (size, demographics, access to contraceptives, etc.) clusters or communities randomly allocated to receive the FH = FW intervention or an attention/time-matched water, sanitation, and hygiene (WASH) comparator intervention. The study took place in a semi-rural district within central Uganda, made up of majority Buganda tribe. In this setting, family planning services, inclusive of individual- and couples-counseling and contraceptive methods, are integrated into general outpatient services and are free at government public health facilities that serve the district. Contraceptives are also available through private not-for-profits and local private shops and clinics for purchase.

Couples were purposively sampled through door-to-door mobilization and through snowball sampling. Couples were eligible to participate if they were: (1) married and lived together most of the time; (2) lived in the selected communities and were available for participation; (3) had an unmet need for family planning (one or both partners in the couple reported wanting to delay pregnancy for at least one year, but were not using modern contraceptive methods); (4) age 18 to 40 for women and 18 to 50 for men or emancipated minors (those under 18 who are married and/or with children who are legal adults in Uganda); (5) of reproductive capability (sexually active in the last three months or planned to resume sex in the next three months if more than one month postpartum); (6) not known to have a medical condition causing infertility; and (7) not pregnant. Men with more than one wife (polygamous couples) were eligible, but only dyads (one man, one woman) were eligible to enroll based on feedback gathered from the community on what would be culturally acceptable [21]. Typically, through door-to-door mobilization, the household visited was the couple recruited, with the wife found at home first and thus, recruited first. However, if the wife did not want to participate, the husband could refer a different wife to the study for participation, if he wanted to. All participants provided written informed consent for participation, and the study was approved by the institutional review boards at the University of Texas at San Antonio in the United States and the Makerere University School of Public Heath in Uganda and by the Uganda National Council for Science and Technology. District leadership permitted entry into the communities. The study’s protocol has been published [36] and the trial was registered with Clinicaltrials.gov (NCT04262882) on February 10, 2020. The pilot trial’s methods have been described in detail elsewhere [33].

### FH = FW intervention overview

FH = FW consisted of four facilitated group sessions with couples (two gender segregated, two gender mixed). The gender segregated groups contained up to 7 individuals, making the maximum 7 couples (14 individuals) in the gender mixed sessions. See Table 1 for an overview of the intervention sessions.

**Table 1:** Overview of the Family Health = Family Wealth Intervention, Uganda 2021-22

Health System-Level	Outlined content
**Health worker capacity building (pre-couple sessions)**	• Family planning refresher training provided to health workers at participating intervention health facility in partnership with the District Health Team to address training gaps in contraceptive knowledge and skills (identified through a needs assessment). Topics covered included information on contraceptive methods (efficacy, side effects, etc.), the technical provision of methods (including practicum-based learning on methods insertion/removal), and the provision of individual- and couples-based counseling.
**Reduced wait time**	• Intervention participants (as individual women or couples) can go ahead in the queue for family planning with study ID card, reinforcing existing “skip the queue” initiatives to incentive men’s engagement in women’s reproductive health services (couples attending together are worked on first), and expanding it by rewarding men’s attendance of family planning community dialogues by allowing women to skip the queue without her partner present if he was part of the group sessions.
**Method distribution**	• Counseling and short-term methods (i.e., condoms, pills), or referral to care for other methods, offered at the end of sessions 3 and 4 from a local health worker.
**Group Sessions**	
**Session 1**	
**Men’s Only Session** *~ 90 min*	• Guided discussion to identify definitions of “family wealth,” barriers to family health and wealth, and redefine group definitions of a “successful” family. Content tailored to analyze and reshape norms relevant to men and women’s separate groups. • Community leader endorses program participation and family planning.
**Women’s Only Session** *~ 90 min*	
**Session 2**	
**Men’s Only Session** *~ 2 h*	• *Relationship Health*: Guided discussion on healthy relationships and family planning (partner violence, communication, decision-making, caregiver roles, gender norms); gender equitable role modeling through vignettes • *Economic Health*: Business skill training co-facilitated with a local business expert to increase interest in the program, improve couples’ shared decision-making, and highlight the importance of planning children to economic health.
**Women’s Only Session** *~ 2 h*	• *Physical Health*: Contraceptive education co-facilitated with a local health worker • *Economic Health*: Business skill training co-facilitated with a local business expert to increase interest in the program, improve couples’ shared decision-making, and highlight the importance of planning children to economic health.
**Session 3**	
Couples’ Session *~ 2 h*	• *Physical Health*: Contraceptive education co-facilitated with local health worker; Health worker provides family planning/linkages to care; couples create a “Family Action Plan” – setting family size and contraception goals • *Relationship Health*: Communication skills building activities; couples set relationship goals in their Family Action Plan (take home assignment) • *Economic Health*: Couples family budgeting; emphasizes the importance of budgeting for the future and highlights the importance of family planning by including the costs of raising children
**Session 4**	
Couples’ Session *~ 2 h*	• *Relationship Health*: Communication skills building activity (health communication/conflict resolution) • Revisit Family Action Plan goals as a couple • *Economic Health*: Couples set economic goals in their Family Action Plan (take home assignment) • Guided discussion to identify community-level barriers and solutions for family planning acceptance, integrated into a “Community Action Plan” co-facilitated with community leader • *Physical Health*: Local health worker to offer family planning/linkages to care • Community leader concludes the program, with final messaging endorsing family planning

Notes: The content is organized by the three areas of “Family Health”: physical health, relationship health, and economic health. There is a total of four sessions, two gender segregated and two gender mixed; All sessions were delivered by two trained intervention facilitators with select content co-facilitated by trained community members (i.e., health worker, local business expert, religious and elected leaders). Sessions took place approximately 1–2 weeks apart from one another

Community dialogues follow a defined process to identify local drivers of sexual and reproductive health with community groups [37] and engage the community in problem-solving towards a common issue through community-based participatory methodologies [38]. Our approach was grounded in Campbell and Cornish’s social psychological theory of transformative communication [29], which emphasizes the role of conversations in safe social spaces in the development of social norms [39]. The dialogue that takes place allows community members to think critically about social norms underpinning a community problem [40], and reconstruct community norms together, creating social environments that promote healthy behavior [41]. The overall goal of our dialogues was to reconstruct individual attitudes and group norms on paths to/definitions of a “successful family” considering three areas of health (physical, relationship, economic) inclusive of family planning. The program also redefined “family planning” as more than contraceptive use but planning for one’s future to ensure health and wellness in all three health areas, which were presented as interrelated and dependent on one another (e.g., one cannot have economic health without relationship health).

Two trained facilitators guided group discussions on the local drivers of poverty as a barrier to family success (with low family planning acceptance emerging as a central driver of poverty among participants). Facilitators also guided critical analysis of known community norms that influence family size expectations and disapproval of family planning locally. The selection of specific norms and beliefs to discuss was informed by earlier qualitative work [11] and multiple, iterative stages of community-engaged research used to develop the FH = FW intervention [21]. The process of selection of these norms/beliefs and detailed examples have been previously detailed [21]. Based on this formative work, we also identified community derived “counters” to gender inequitable or otherwise “unhealthy” norms that align with the program’s definition of family success and the use of family planning, which facilitators were trained to lead discussions towards. Since counters were derived from the community, the goal was for counters to be natural conclusions for participants to come to on their own. For example, men and women each considered the idea that having many children is tied to being perceived as a “real man” or a “real woman.” Facilitators led couples through discussion on whether this belief could affect other aspects of being a man or woman that were important to them, such as achieving other goals for their families and themselves. The final reshaped belief that facilitators aimed to elicit from participants was the idea that strict adherence to traditional gender roles is not always beneficial for a family. For men and women’s separate group sessions, dialogues were tailored to include norms most relevant to their barriers to family planning.

In addition to the dialogues, the group sessions also included content to target individual, interpersonal, and community drivers of an unmet need for family planning guided by the social ecological model [30–32]. As outlined in Table 1, this content included individual and couple-based education, skills-building, and goal-setting activities, community leader involvement, and the development of a “Community Action Plan” with group-derived solutions to identified barriers to family success, all designed to reinforce family planning’s benefits to family success in the three areas of health. This content included gender transformative approaches to reinforce the importance of gender equitable relationships on family health and build necessary skills in gender equitable communication and shared decisions-making (e.g., gender equitable couple modeling through vignettes/role-plays, communication skills workshops, and couples’ goals setting within the Family Action Plans). The content was developed and tailored for men and women’s groups based on a pre-intervention needs assessment, which found men needed to be sensitized on relationship issues before meeting with women (resulting in men’s session 2 relationship content), and suggested that too much family planning content might make men lose interest in the program (resulting in the decision to have two family planning focused sessions for women and one for men) [21]. Following local customs for community meetings, participants received 5,000 Ugandan Shillings as a transport reimbursement for attendance to each meeting and light refreshments were served.

Group sessions were also paired with health system strengthening elements aimed to reduce access barriers. In brief, we offered couples the ability to opt-in after sessions 3 and 4 for couples-based family planning counseling with the health worker and to receive either short-term methods or referral to care for other methods. We provided a refresher training on the provision of individual- and couple-based family planning counseling to improve provider capacity to deliver all methods. Finally, we expanded on existing incentives for men’s engagement in family planning services that allow couples to be worked on first if they come for family planning services together by allowing women to be worked on first if they present to care with their study ID card. This served to similarly reward men’s engagement in family planning dialogues without requiring their clinic attendance, although it was still encouraged.

### Data collection methods

The FH = FW intervention arm included 35 couples (*N* = 70) with an unmet need for family planning; characteristics of the sample have been reported in detail elsewhere [33]. To summarize, participants in the FH = FW arm were 29 years of age on average (SD = 6.8), mainly from the Buganda tribe (87.1%), and approximately half Catholic/Protestant/Other (51.4%) vs. Muslim (48.6%) (mirroring the make-up of the region). All couples were married and living together and had been together for an average of 6.2 years (SD = 5.7, range 0–21 years). The average number of children per couple was 2.5 (SD = 2.0), and 14.3% of couples were in a polygamous marriage. At baseline, 42.9% of participants said they had used modern contraceptives in their lifetime.

Participants were followed for approximately ten months, with quantitative data collected at baseline, and approximately seven and ten-months post-enrollment. There were two points of qualitative data collection in this timeframe (used in the present paper). All FH = FW participants were invited to: (1) an in-depth, one-on-one interview approximately three weeks after all intervention sessions were implemented, and (2) either a focus group discussion or individual interview again after the 10-month follow-up quantitative data collection. For the interview approximately three weeks post intervention, semi-structured, in-depth interviews were conducted individually with 64 out of the 70 participants (91% of intervention sample). The structured interview protocol elicited participants’ experience with the intervention and its potential positive, negative, and null effects on family planning, relationship, economic, and other relevant outcomes. Interviews lasted approximately 45 min and were facilitated in Luganda (local language) by an experienced qualitative interviewer at the participants’ home or a place of their choosing or over the phone for COVID-19 risk mitigation (9 out of the 64 interviews [14%] were over the phone).

For the final qualitative assessments after the 10-month follow-up, we used the quantitative data measuring contraceptive uptake to divide participants into two separate groups: focus group discussions with participants who reported contraceptive uptake during the study period, and one-on-one, semi-structured interviews with those reporting no contraceptive uptake. Participants were separated into these two different methods, so that reasons for non-use could be explored in more private one-on-one interviews. Of the total sample, 94% (*n* = 66/70) of participants participated in either the final exit interview or focus group. An experienced facilitator conducted 5 focus group discussions (2 with women, 3 with men) (*n* = 39) in Luganda in a community-setting, with a research assistant taking detailed notes. Focus groups were approximately 90-minutes. An additional 27 one-on-one semi-structured, in-depth interviews were conducted in Luganda by a trained qualitative interviewer (~ 20-minutes in duration). The interview guides had similar questions as the three-week post-intervention interview, with the addition of questions to explore if participants’ family planning desires changed from baseline and why they were not using contraception (for individual interviews). Some of the interviewers were involved in the study as intervention facilitators, as they had built rapport with the participants and had a strong understanding of the FH = FW program, allowing them facilitate in-depth discussions related to FH = FW’s content. All focus group discussions and individual interviews were audio-recorded, translated to English, and transcribed.

### Data analysis approach

Data were analyzed thematically [42]. The project directors (KMS, CM, SMK) iteratively reviewed the transcripts to develop a coding guide structured around identifying intervention effects or null effects within the intervention’s three areas of health. Most relevant to the aims of this specific analysis was intervention effects on norm/belief transformation. Trained research assistants used an iterative process to manually apply codes to transcripts, meeting weekly with KMS to discuss and resolve discrepancies, who reviewed all excerpts after data were fully coded for consensus or re-coding; codes were organized in an Excel spreadsheet. Data from the two separate time periods and methods (focus groups and interviews) were triangulated by ensuring codes were built off of data from different individuals and from both data sources (36). KMS, CM, and SMK also reviewed data from contraceptive non-user interviews and contraceptive user focus groups (10-months post-intervention) for similarities and differences to validate the data, while seeking to identify if there were thematic differences between these groups. Codes that represented thematic elements were collated and themes with representative quotations were summarized by KMS and DT. While Campbell and Cornish’s social psychological theory of transformative communication [29] does not have specific constructs to structure a coding guide, the theory as a whole guided the review of data and identification of themes related to normative change.

## Results

We identified five key themes that demonstrate synergies between FH = FW’s holistic and community dialogue approach in challenging inequitable gender norms and other community norms, increasing gender equity within couples, and ultimately increasing acceptance of family planning as described in detail in the next sections.

### Theme 1: community family size expectations were reconsidered through discussions on changing economic conditions, the cost of raising children, and child development

The FH = FW program reiterated what participants already knew about the intersection of economic health and family planning (gleaned from our earlier work that informed the intervention) – that having more children than one can manage can have negative economic consequences for families and is a source of poverty locally. It was apparent from the data that the program deepened participants’ understanding of this through facilitated group discussions critically analyzing the sources of poverty locally alongside community and religious norms on family size expectations (e.g., the expectation to have as many children as one’s father, for men to “expand the clan,” and for men to not limit the number of children they have). Many participants came to their own conclusions that traditional expectations about family size do not align with what can be managed today, translating to changed attitudes towards family planning, as one man (age 31, ID 1) said during a focus group discussion (10-months post-intervention), “*At the end of the day, the burden is upon your shoulders to feed and take care of the children – not religion or culture*.” The following quotation also demonstrates how guided critical analysis of cultural expectations for large family size facilitated the realization that these expectations are no longer realistic in the context of current economic conditions, which increased participants’ acceptance of family planning. This was a salient finding among men in particular. The same man goes on to say:

*“Personally, my grandfather gave birth to 100 children, my father to 50 children. So, we had a mentality as we were growing up that we should also emulate our forefathers and, personally, I wanted to have a minimum of 25 children and a certain brother of mine wanted 40 children. After this program, I have changed completely. I even think if I give birth to two children, as long as I can provide for them well and see them through school, that is enough for me”* (Man, age 31 [ID 1], 10-months post-intervention focus group).

For some participants, these changing ideals on family size were also apparent for expectations originating from religious norms. These findings are in the context of a community with a large Muslim population that practices polygamy, but where the practice of polygamy and ideals of having children from multiple wives is held widely among men regardless of religious background. Notably, in community dialogues, when discussing religious expectations to not limit the number of wives and children that men have, many participants often countered this (aligning with the intervention counter belief), stating that the Koran emphasizes that men should only take on more wives when they can provide for the one(s) that they already have. As the quotation from one man who participated in the intervention showcases, the intervention message to limit children for economic reasons was received well by most men.

*“We are living in another era where so many things, as you taught us, have changed – the land is smaller, the resources are limited, the cost of living has gone up, and I noticed that I don’t have the capacity any more to afford a large family size. I noticed that I cannot afford to fulfill what religion demands”* (Man, age 31 [ID 2], 10-months post-intervention focus group).

One woman similarly expressed how the group dialogues made her reconsider family size expectations against changing economic conditions: “*From the study, we saw that even the belief ‘every child comes with their special blessing’ does not count any more in this era, where economic prices have skyrocketed and every child demands lots of things to make them successful*” (Woman, age 28, 10-months post-intervention focus group). The belief in reference is a commonly held belief locally, that one must have many children (and children from multiple women, for men) to increase the chances or “luck” of having a successful child. The above quotation demonstrates not only the participant’s reconsideration of ideal family size against economic conditions, but also the reassessment of the belief of “lucky” children and subsequent adoption of the programs’ “counter” belief (intervention counter belief: child success and development comes from personal investments, such as education, good nutrition, healthcare, personal time/attention and this investment can be better achieved when children are planned for and spaced). When discussing beliefs that were reconsidered based on the intervention in the exit focus group, the “lucky” child belief was cited by multiple participants (majority men).

Across narratives, including both contraceptive users and non-users at 10-months follow-up, participants expressed an increased understanding of the link between economic health, family success, and family planning. This was evidenced by the different economic goals they set and the related practices that they adopted as couples since the program. All participants reported working on economic goals based on the intervention (e.g., working towards savings goals, starting a small family business, using the family budget provided by the program). When participants described these goals and practices, many of them commonly spoke of them alongside family planning, the cost of raising children, and the importance of timing and spacing children for economic well-being. The following experience shared by one woman (age 24, 10-months post-intervention focus group) is a representative example of how couples began to consider family size in relation to economic goals: “*Goal setting made us realize that it is not a matter of just producing children when you have not planned how you will provide for them. After deciding on the number of children to have and when to have them, we had to consider our savings and spending*.”

### Theme 2: showcasing how relationship health and gender equity are central to economic health influenced men’s acceptance of more gender equitable attitudes and relationship dynamics

The qualitative data demonstrates that both men and women acquired new knowledge on the bidirectional relationship between healthy relationships and economic wellness. Participants shared that they learned that having a strong relationship with their spouse could have positive effects on their economic standing, by improving their partnership in financial planning, developing or growing a family business, and working towards shared savings and economic goals. In the intervention, local business experts and program facilitators encouraged couples to consider starting (or growing an existing) family business for additional revenue, and shared their own experience as a successful community role model. Examples were activities common in the setting that required low start-up capital, and were tailored towards what men and women typically do for their sessions (e.g., sustenance farming, trade, and small businesses). In addition, couples participated together in learning to make a family budget and set financial goals. As one man (age 50, 10-months post-intervention focus groups) explained: “*We were taught to have a family budget and to also save. If we budget together and save together, there is financial trust being built and it doesn’t end at that. Therefore, if we [as a couple] are one [in agreement], then family planning [broad program definition] comes easily, because we have certain targets we want to meet.”*

As illustrated by this quotation, understanding the importance of a healthy spousal relationship to economic health worked to create motivation for healthier, more equitable relationships, making economic health content an entry point for more gender equity. This was especially salient in men’s interviews and focus groups (for contraceptive users and non-users alike), who expressed a strong desire to improve their family’s economic standing. However, there were limitations for some couples in what was considered acceptable in terms of the integration of couples’ finances and business activities. A subsample of participants explicitly stated that if they already had their own income generating activities before the study, they did not want to involve their spouse, but were open to supporting each other in starting their own new businesses. Other narratives, from both women and men, suggested some relationships needed further strengthening for couples to successfully work together on the economic wellness aspects of the study. As one woman (age 25) stated, “*The family budget is good, but realistically it cannot work well, reason being men cannot fully tell you their earnings. You can save together but he will keep some money to himself. You too will do the same and at the end it is not so effective*” (10-months post-intervention focus group).

However, the majority of participants’ narratives suggested that the gender transformative elements of the group sessions and dialogues helped them to understand how equitable relationships (e.g., those with shared decision-making, free of violence) aided in their family’s economic and other goals. Dialogues also helped participants think about traditional gender roles and the division of labor, where men work and are the primary earners and women raise children and are responsible for household chores, and consider whether strict adherence to such roles resulted in the best outcomes of family success. These discussions, paired with opportunities for couples to practice equitable decision-making in the development of their economic goals (through the Family Action Plan), seemed to contribute to reduced gender inequity in couples’ economic lives and their division of labor in many couples. Narratives from all focus groups and many interviews with men, with confirmation from women’s narratives, include examples of men changing their minds about women’s participation in household decision-making and non-traditional work and earning. In turn, in women’s narratives included expressions of empowerment.

“*Whenever it came to taking major decisions, like business, schools for our children, and any major developments, I would not see the reason to consult with [my wife] since she does not work, but is just a house wife. I would prefer to go back to my parents and get advice from them. But after the training, this changed now and we are on a journey of setting up a successful home and working on our lives*” (Man, age 27, 10-month post-intervention focus group).

“*From the sessions, I learned that it is not only a man who can make decisions. Even we women can have some brilliant ideas to share, and we were encouraged to never keep quiet”* (Woman, age 27, 10-month post-intervention focus group).

Some participants explicitly described the positive effects that increased economic and decision-making equity had on themselves, their spouse, and the quality of their relationship. For example, a few men noted how women’s participation in earning helped reduce the burden placed on men by gender role expectations to be the sole breadwinner (gender role strain):

*“I am a farmer while my wife has a business that I started with her. We discussed about it and made the conclusion that, even if I am a farmer, we should at least have a savings scheme at home whereby I can at least bring 2,000 [Shillings], she also brings 2,000 [Shillings] per day and we’ll save. And we develop ourselves”* (Man, age 31 [ID 3], 3-weeks post-intervention interview).

Some men also noted increased harmony with and satisfaction among their wives due to this shift towards more equity, and a portion of women and men (those where the woman had newly begun working since the intervention) described how reduced economic stress resulted in less conflict overall, as one woman (age 31) explained in the focus groups (10-months post-intervention): “*Surely, when I did [begin working], I realized that conflicts stopped in our home, because I work as well as my husband, so we put funds together at the end of the day, and plan for the family.”* These positive experiences reinforced the intersection between economic health (and equity) with relationship health, increasing motivation for continuing to practice more gender equitable relationships.

### Theme 3: linking relationship health and family planning helped increase positive attitudes towards family planning and the perceived importance of shared household decision-making to family wellness

Participants shared that a common outcome of intervention participation was gained knowledge on how stress negatively affects relationships, including the stress of having more children than one can manage (and related financial strain). For example, in a focus group (10-months post-intervention), one woman (age 26) shared her understanding of how a lack of family planning can hurt relationship satisfaction, a perspective repeated by multiple women, “*When you give birth to too many children, it kills all the love in the family, and especially from your husband*.” In addition, a few narratives from men revealed that the intervention successfully dispelled or reconstructed commonly held misconceptions for some participants related to relationships and family planning (an aim integrated into group dialogues and vignettes, informed by our formative work) – like the gender inequitable belief among some men that if married women are using family planning it means they are unfaithful. As one man (age 30) shared in the focus group (10-months post-intervention):

*We had a mentality that if a woman uses family planning, she is sexually immoral. If she is married and uses it without the husband’s knowledge, then she is an adulteress. This would be a point of contention in so many peoples’ homes. After attending this program, we realized that for a woman to use family planning, it is not necessarily that she is immoral; there are so many reasons for using [family planning].*

In women’s focus groups and interviews, there was also evidence of change in the belief that having a child to please one’s husband helps relationships and can fix relationship problems, as one woman (age 19, 10-months post-intervention focus group) said: “*I thought that the more children you give birth to, the more love the man gives you, but from the program, I learned that the man’s love doesn’t depend on that*.” For a subgroup of participants who held this belief and struggled with relationship challenges before the intervention, this new knowledge served to reduce negative attitudes about family planning.

In men’s sessions, the intervention content aimed to link relationship health to family planning by helping men understand how role neglect was negatively affecting their relationships and adding to their economic stress by taking on more wives/children than they could cater for. The facilitator guided discussions on how seeking other relationships and having children with other women in response to dissatisfaction with their current relationship was harmful to men and families both economically and in their relationship health (a commonly cited relationship problem from women). FH = FW’s “counter” conclusion that we intended men to come to was that they should work on improving their current relationships and fulfilling their current roles. However, in focus groups and interviews, men did not discuss this content or cite it as motivating them to change their relationship practices or their ideas about family planning, suggesting that this portion of the intervention may not have been influential for men.

Most salient in participants’ narratives about the link between relationship health and family planning was increased knowledge that health-related decisions, including decisions on family planning, should be made together. As one man (age 28) stated in focus groups (10-months post-intervention), “*In your training, you taught us a lot about the advantages of working and planning together*.” Participants’ changed attitudes for shared decision-making on health issues, including family planning, was rooted in the desire to increase physical health and well-being, as well as harmony within relationships, informed by new knowledge gained on the intersections of these concepts. However, it was apparent that a subsample of participants were still struggling with relationship issues by the end of the study, who were contraceptive non-users at 10-months follow-up, and harmonious decision-making as a result. These participants are discussed in more detail in the next theme.

### Theme 4: program elements to strengthen relationship skills helped to translate more gender equitable attitudes into changes in relationship dynamics and to facilitate equitable family planning communication

A salient theme across men and women’s narratives was that the specific relationship health program elements (i.e., communication skill-building activities; family planning, economic, and relationship goal setting through the Family Action Plan; gender transformative dialogues and vignettes focused on relationships and equity) were successful in their overall aim to develop relationship skills and to further reduce gender inequitable attitudes. The majority of narratives confirmed that these skills were lacking and were a consistent source of stress within couples before the start of the study. Many narratives confirmed that developing relationship skills was necessary to facilitate conversations about the three areas of health and the couples’ family goals, and to increase men’s willingness to engage women in household decisions equitably. One man reflected on how increasing communication skills generally between couples translated into improved communication about family planning.

*[The program] addressed the barrier of poor communication. The communication between me and my wife was poor; I could not tell her my problem and she too could not tell me my mistakes. So, we were in this kind of situation that we could not communicate well. If we failed [to communicate] on small matters, then how could we [communicate] on sensitive issues, like family planning? This is to say, if communication is okay in couples, then family planning can easily be adopted* (Man, age 28, 10-months post-intervention focus group).

This participant’s narrative also highlights the gender inequitable attitudes that had to be reconstructed through this process to change relationship dynamics. These dynamics, of men having more power within relationships and decision-making, and a lack of open and constructive communication between couples (particularly from wife to husband) align with the broader cultural norms of the community dictating inequitable communication between men and women. The next quotation (representative of the majority of participants who expressed positive changes in this area) further illustrates the interplay of changed gender equitable attitudes, increased communication and decision-making skills, and increased spousal agreement in using family planning through collective goal-setting.

*Before we were trained on relationship health, our spouses were still holding onto the cultural values on how men should not listen and agree with their wives. After learning, they are now in agreement and consultation with us. We even managed to set our goals on child timing and spacing. They themselves walked us to the health facilities to receive the contraceptive methods. The barriers are no longer there* (Woman, age 24, 10-months post-intervention focus group).

The narratives suggest that having facilitators guide couples through this process, paired with couples-based activities like the Family Action Plan (couple goal setting), allowed participants to develop and practice more gender equitable interactions in a safe space with support. A common suggestion from participants was for more time to be spent on relationship strengthening or the addition of one-on-one couples’ counseling, demonstrating a need for this content for couples. Moreover, while no participants explicitly expressed resistance towards gender equitable relationships or direct opposition to changing traditional gender community norms, there were a few couples who participated in individual interviews (i.e., the group whose unmet need for family planning remained at the end of the study), who expressed continued conflict within their relationships to the point of separation or a complete breakdown of communication. For these couples, they stated that the intervention’s relationship content was not enough for them to overcome their challenges (e.g., infidelity expressed in one couple), and that they needed additional, one-one-one support and follow-up outside of the study.

### Theme 5: participation in FH = FW increased collective decision-making about family size goals, desired pregnancy timing, and uptake of contraceptive methods

Taken together, the majority of participants described adopting more gender equitable attitudes due to FH = FW participation, and in many couples, this increased women’s decision-making power within the relationship overall, inclusive of health and family planning decisions. Narratives suggested that most decisions about family planning were made collaboratively between partners, which was the aim of the program. For most couples, reproductive decision-making made exclusively by men, or by women in secret (which was reportedly common in the sample and overall community), was reduced. For example, one woman stated, “*After the program, both of us agreed on the choice of family planning method. I am using the Norplant, and it has given us a break. It is soon expiring, but I plan to use it again”* (Woman, age 23, 3-week post- intervention interview).

There were also clear examples in non-contraceptive user interviews of increased openness to using contraceptives in the future, but shared decisions to not use contraceptives yet based on couples’ shared pregnancy desires. For example, one man stated (age 31, ID 4, 10-month post-intervention interview), “*We desire to have five children and we agreed together on it. But we can’t start using a family planning method when we have not had a child yet.”* These findings align with person-centered goals of family planning programs, that include not only contraceptive use but the informed and autonomous decision to not use contraceptives.

It is important to note, however, that there were also narratives suggesting that, in some couples, men still ultimately held the decision-making power, for example, one woman (age 24) said in the exit focus group (10-months post intervention): “*[The program] helped my husband so much, because he is the one who chose for me the method to use of his preferred choice. Actually, it made me feel happy.”* In these cases, women expressed their own desire to use family planning (reproductive autonomy), and their partner’s desires aligning with their own through the program’s positive impact on men’s motivation for family planning. However, these narratives also suggest that the husband’s desire for family planning and contraceptive preferences may still weigh more heavily than women’s desire.

## Discussion

This qualitative evaluation of Family Health = Family Wealth (FH = FW), a community-based, couples’ intervention, supports positive effects of using gender transformative community dialogues paired with other multi-component content to increase couples’ acceptance of family planning in rural Uganda. Grounded in Campbell and Cornish’s social psychological theory of transformative communication [29], qualitative data collected from men and women at two time points post-intervention provides support that the community dialogue approach helped couples to adopt new beliefs about family health and success, while reassessing previous community beliefs that reinforce gender inequity and disapproval of family planning, and consider their ideal family size in relation to their family goals. The findings suggest FH = FW’s holistic approach and inclusion of economic and relationship content was highly engaging to participants, and the relationship health and economic health content served as key entry points for couples to discuss family planning, as well as opportunities to build relationship skills relevant for equitable communication and decision-making. Taken together, FH = FW’s holistic approach was key to creating the conditions in which norms/beliefs could be reshaped through transformative communication and new norms formed around alternative definitions of family success.

While community dialogues have been widely used by civil society, bilateral, and multinational agencies for reproductive health [43], they have been less commonly rigorously tested and published in peer-reviewed literature [44]. Of those that have been published in the peer-reviewed literature, there is evidence of their ability to influence normative change; studies in sub-Saharan Africa report positive effects of community dialogue participation on reducing HIV stigma, increasing gender equitable attitudes, and increasing community ownership of a problem [44–46]. This study adds to this body of evidence, suggesting this approach can be influential on individual endorsement of community norms associated with family planning. It is worth noting, however, that facilitators of the intervention expressed that the discussions on normative change took time, and were sometimes met with initial resistance (to be detailed in a separate manuscript focused on program implementation). Thus, transformative dialogues require well-trained facilitators, time, and multiple opportunities to reinforce normative change. Importantly, FH = FW’s content was developed through community-engaged research methods conducted with the local community [21], and community-based participatory methods common to the community dialogue approach were used to help guide participants towards the shaping of community norms towards “counter beliefs” that were derived from the community itself [38]. This approach is important in the building of family planning dialogues that appropriately address local norms and beliefs. The qualitative data presented here suggest FH = FW’s selected norms were culturally relevant drivers of family size and family planning acceptance locally that could be reshaped towards gender equity and family planning acceptance.

This study adds to a growing body of evidence that supports the importance of integrating gender transformative content into interventions with individuals, couples, and at the community-level in order to positively impact sexual and reproductive health outcomes [47–50]. Our qualitative findings suggest that the dialogues helped men to move away from traditional beliefs around gender roles and norms and recognize the benefits of increasing equity within their relationship. Couples’ relationships improved as a result, with subsequent impacts on men and women’s ability and willingness to discuss and make family decisions together (including those relating to family planning). The broad framing of our intervention around family health and wellness helped to engage men in the intervention and create the conditions for men and women to adopt more gender equitable attitudes and relationships. This approach is something that can be adopted by other couples-based group interventions aimed at increasing family planning through gender norm change. Our findings also suggest that gender transformative dialogues are likely more effective when paired with activities to help couples build skills to help facilitate the translation of changed attitudes into changed relationship dynamics, as FH = FW did (e.g., the Family Action Plan [family goals], Community Action Plan, budget activity). However, for several couples facing deeper relationship issues, they expressed the need for more direct relationship support beyond what the group sessions offered. Finally, while our study provides evidence that FH = FW helped to increase women’s reproductive autonomy, our finding that some men still seemed to hold the final decision-making power around contraception choice in our sample is evidence of how deeply engrained these gender dynamics are, and the need for continued work in this area.

This qualitative analysis allowed us to explore potential changes in endorsement of local community norms, family planning acceptance, and family planning behavior associated with FH = FW participation. However, the limitations of this analysis should be made explicit. This qualitative data is not a direct test of these relationships or behavior change. However, the findings do align with our previously reported quantitative findings; in the larger pilot trial, there was 31% more contraceptive uptake in the FH = FW arm at 7-months follow-up and 40% more at 10-months follow-up compared to an attention-matched comparator intervention [33], as well as positive change in other family planning determinants that were quantitatively assessed (e.g., contraceptive knowledge, family planning intentions) [34]. Most relevant to the current paper’s qualitative findings, the study’s larger findings specifically supported increased positive attitudes towards family planning, reduced gender inequitable attitudes, and positive perceptions of family planning norms (perceived acceptance of family planning among others) in FH = FW participants compared to those who received the comparator intervention [34]. Improvements in relationship dynamics were also supported by the quantitative data, including increased spousal family planning communication and joint household decision-making [34]. While these findings from the larger study help to validate the qualitative data presented in this paper, they are also limited by the pilot trial’s quasi-experimental methods and sample size (described in detail elsewhere) [33]. Thus, FH = FW needs to be evaluated in a full efficacy trial in which its effect on contraceptive uptake and related outcomes can be established and the relationships proposed in this study fully tested.

Social desirability could have influenced participants’ responses, especially since participants reported little dissatisfaction with, or unintended negative effects of, the intervention. The fact that some interviews were conducted by individuals associated with the study increases the risk that participants’ responses were influenced by social desirability bias. While there were no continued or planned project activities after the completion of the study, participants expressed the desire for the project to come back. The desire to receive future benefits from the study could have also influenced their high reporting of satisfaction with the program.

It is also worth emphasizing that this paper aimed to explore the effect of the transformative community dialogues on the reshaping of personal endorsement of community norms related to family planning and subsequent effects. A future, larger scale test of this intervention could examine its actual effect on community norms (rather than individual attitudes). Others point out that more evidence needs to be gathered to demonstrate that gender transformative interventions can have a sustained effect on broader norm change [51]. Further, there are other elements of the intervention that likely influenced attitude and behavior change as well that were not explicitly highlighted in this paper (e.g., education on contraceptive methods). Thus, the findings highlighted in this paper are not exhaustive of the potential pathways between our intervention and the decision to use family planning. Findings related to other multilevel intervention components have been published elsewhere [34].

Despite these limitations, this qualitative exploration provides support for the FH = FW intervention’s ability to change individual endorsement of widespread community norms. The findings underscore the importance of addressing gender and related cultural norms that reduce family planning acceptance, while promoting family planning’s holistic benefits inclusive of physical, relationship, and economic benefits that are of high importance to couples. Family planning interventions like FH = FW that explore innovative ways to engage men and create more gender equity within couples are needed and could make a large impact on reducing the unmet need for family planning in Uganda and similar settings. Future work will aim to reproduce and expand on these findings by testing FH = FW in a larger community efficacy trial, while gathering data to support the feasible and cost-effective scale up of this approach.

## Data Availability

The qualitative data reported in this paper will not be shared due to ethical concerns regarding the privacy of the participants.
